# Oxidative Stability, Microbial Safety, and Sensory Properties of Flaxseed (*Linum usitatissimum* L.) Oil Infused with Spices and Herbs

**DOI:** 10.3390/antiox10050785

**Published:** 2021-05-15

**Authors:** Dyana Odeh, Klara Kraljić, Andrea Benussi Skukan, Dubravka Škevin

**Affiliations:** 1 Division of Animal Physiology, Faculty of Science, University of Zagreb, Rooseveltov trg 6, 10000 Zagreb, Croatia; 2 Department of Food Engineering, Faculty of Food Technology and Biotechnology, University of Zagreb, Pierottijeva 6, 10000 Zagreb, Croatia; kkraljic@pbf.hr (K.K.); dskevin@pbf.hr (D.Š.); 3 Food Control Center, Faculty of Food Technology and Biotechnology, University of Zagreb, Jagićeva 31, 10000 Zagreb, Croatia; abskukan@pbf.hr

**Keywords:** flaxseed oil, oxidative stability, spices/herbs, natural antioxidants, microbial food safety, sensory analysis

## Abstract

In our study, we assessed whether the addition of basil, fennel, oregano, rosemary, and chili can improve oxidative stability and sensory properties of flaxseed oil (FO) during 180 days of storage or induce oil contamination by microorganisms. Results showed that addition of spices and herbs in FO affected the hydrolytic changes, but far less than 2% of free fatty acids after storage, which was in line with regulations. Further, the addition of spices and herbs in FO decreased peroxide value (even up to 68.7% in FO with oregano) vs. FO whose value increased during storage, indicating increased oxidative stability and prolongation of shelf life of infused oils. The antioxidant activity of the infused oils ranged from 56.40% to 97.66%. In addition, the phenol content was higher in all infused oils (6.81–22.92 mg/kg) vs. FO (5.44 mg/kg), indicating that herbs and spices could scavenge free radicals and inhibit lipid peroxidation, while sensory analysts showed that FO infused with chili had the lowest bitterness intensity. According to the presence of certain microorganisms, results highlighted the need to develop new methods for inactivating microorganisms that would not only provide a microbial safety, but also preserve the beneficial properties of the oils/products.

## 1. Introduction

Flaxseed oil is well known for its positive impact on human health, mainly attributed to its high content of essential omega-3 α linolenic acid (ALA), which makes up more than 50% of total fatty acids [1]. During storage, flaxseed oil is subject to changes that diminish the quality of produced oil. Hydrolytic changes (lipolysis) are mostly influenced by moisture, temperature, and the presence of enzymes and microorganisms [2], while the oxidation process causes destruction of some biologically active compounds, such as unsaturated fatty acids and vitamins. ALA oxidizes 20–40 times faster than oleic acid and 2–4 times faster than linoleic acid [3]. Therefore, flaxseed oil has a limited shelf life that can be even shorter due to chemical reactions, as well as enzymatic and microbiological processes, if the storage and handling are not adequate [4]. That leads to a loss of nutritional value, alters the physicochemical properties of oils, reduces shelf life, and gives an undesirable odor and taste to the oil [5,6]. Based on the above, the limited shelf life of the oil can be improved by the addition of natural antioxidants.

Today, a number of compounds are known to have antioxidant properties, but only some of them can be used to prolong the shelf life of oils, since the selection of natural antioxidants to prevent lipid oxidation is challenging and depends on multiple factors such as fatty acid profile of the oil, minor components present in the oil, and structure of the antioxidants [6,7,8,9]. The antioxidant activity of spices and herbs has been increasingly investigated, and food manufacturers are trying to replace synthetic antioxidants with natural compounds, which, in addition to slowing oxidation processes, also have a positive effect on consumer health and good image among consumers [10,11,12]. Some natural antioxidants; i.e., plant-derived compounds such as carnosol, rosmanol, rosmariquinone, and rosmaridiphenol, may be up to four times as effective as butylated hydroxyanisole (BHA), and equal to butylated hydroxytoluene (BHT) due to synergism with other preservation methods, and are also reported to be superior in terms of safety, prevention of nutrition-related diseases, and improvements of physical and mental well-being of consumers [7,10,13,14,15,16,17].

In Mediterranean cuisine, the addition of various spices and herbs to oils, especially olive oil, is a traditional practice to enhance the taste and aroma of the dish, as well as for food preservation. For these preparations, so-called “infused oils”, various herbs and spices like rosemary (*Salvia rosmarinus* Spenn.), oregano (*Origanum vulgare* L.), basil (*Ocimum basilicum* L.), fennel (*Foeniculum vulgare* Mill.), and chili (*Capsicum* spp.) are most often used because of the climate, and the presence of numerous valuable compounds (phenolic compounds, vitamins C and E, glutathione) that show specific properties, such as antioxidant, antidiabetic, antimutagenic, antitoxigenic, antimicrobial, and others [13,18,19,20,21,22,23,24].

Further, herbs and spices do not normally raise health concerns in humans, but they can be contaminated with microorganisms that can cause spoilage, whose growth during food storage increases or may allow the transmission of pathogenic bacteria, such as *Salmonella* spp., *E. coli*, *Campylobacter,* and *Shigella* spp., through the feces of birds or animals or by *Clostridium botulinum* present in the soil, which can be dangerous for the consumer [25,26]. This should not be a problem unless processing or storage conditions have been such that they allowed subsequent spore germination, growth, and formation of toxins. Consumption of microbe-contaminated food, as well as their toxins, can lead to consumer poisoning and endanger the health of consumers, so it is important to keep in mind this biological hazard to prevent contamination of products at an early stage of production and ensure microbial safety [27].

Therefore, the aim of this study was to increase the oxidative stability of flaxseed oil by using natural antioxidants such as rosemary, oregano, basil, fennel, and chili based on beneficial health effects and the Mediterranean climate, as well as to evaluate the sensory properties of flaxseed oil infused with different spices and herbs. In addition, regarding the mentioned potential biological hazards that herbs and spices bear, microbiological testing of infused oil was also carried out to determine whether added herbs or spices contaminated the oils.

## 2. Materials and Methods

### 2.1. Materials

Eco cold-pressed flaxseed oil was obtained from local producer Janković (Unčani, Dvor, Croatia). Oil was freshy produced and immediately used for infused oil preparation. For preparation of infused oils, dry plant materials—basil (*Ocimum basilicum* L.), fennel (*Foeniculum vulgare* Mill.), oregano (*Origanum vulgare* L.), rosemary (*Salvia Rosmarinus* Spenn.), and chili (*Capsicum* spp.) were obtained from local distributer Suban (Strmec Samoborski, Croatia).

### 2.2. Reagents and Standards

All chemicals and solvents were analytical grade and obtained from commercial sources. The polyphenolic components (gallic acid, 3,4-dihydroxybenzoic acid, 4-hydroxybenzoic acid, tyrosol, chlorogenic acid, 2,4-dihydroxybenzoic acid, ferulic acid, *p*-coumaric acid, *o*-coumaric acid, sinapic acid, vanillin, secoisolariciresinol diglucoside (SDG), syringaldehyde, oleuropein, syringic acid, *trans*-cinnamic acid, and vanillic acid), as well as the 2,2-diphenyl-1-picrylhydrazyl radical (DPPH), were all purchased from Sigma-Aldrich Co. (St. Louis, MO, USA). Anhydrous sodium sulphate was purchased from Kemika d.d., Croatia; methanol, formic acid, and sodium hydroxide from Carlo Erba Réactifs-SdS, France; phosphoric acid from Fluka, Switzerland; and acetonitrile, n-hexane, diethyl ether from J.T. Baker, Netherlands.

### 2.3. Preparation of Flaxseed Oil Samples Infused with Herbs and Spices

Infused oils were prepared at a concentration of 5 g of spices or herbs per 100 g of oil according to the method described by Škevin et al. [28]. The finely ground spices and herbs were added to coldpressed flaxseed oil and macerated on a laboratory shaker at 140 rpm for 48 h at 25 °C. After 48 h, the samples were filtered through a 11 µm pore size filter paper using a Büchner funnel and vacuum. The infused oils were stored in dark glass bottles under nitrogen at a room temperature until analysis. Freshly prepared oils were tested, and also after 2, 4, and 6 months of storage to assess the oil quality, microbial safety of the oil, oxidative stability, and sensory quality.

### 2.4. Determination of Oil Quality Parameters

Free fatty acids (FFA) and peroxide value (PV) were determined using standard ISO methods. The content of FFA was determined according to the ISO 660 [29], and the results are expressed as % of oleic acid. To determine FFA content, 10 g of an oil sample was weighed in a 250 mL Erlenmeyer flask and then dissolved in 50 mL of a previously neutralized mixture of diethyl ether (peroxide-free) and ethanol (96%) at a ratio of 1:1 (*v*/*v*) to dissolve the test portion. The prepared solution was titrated with 0.1 mol/L sodium hydroxide until the color of the added indicator, phenolphthalein, changed. PV was determined according to the ISO 3960 [30] standard method, and the results are expressed as mmol O_2_/kg. Briefly, 5 g of the oil was dissolved in 50 mL of a previously prepared solution of isooctane and acetic acid at a ratio of 2:3 (*v*/*v*). Then, 0.5 mL of saturated potassium iodide solution was added and mixed for exactly 1 min. The reaction was quenched by the addition of 100 mL of water, and liberated iodine was titrated with 0.01 mol/L sodium thiosulfate solution. A volume of 0.5 mL of 1% starch solution was added as an indicator.

### 2.5. HPLC Analysis of Polyphenolic Compounds in Oil Samples

The determination of the content and composition of polyphenols was performed according to the method described in Kraljić et al. [31]. Exactly 4 g of the infused oil sample was weighed in a tube and 5 mL of 80% methanol was added. The samples were mixed in a vortex for 1 min. The tube was then placed in an ultrasonic bath for 15 min, and samples were centrifuged at 4000 rpm for 20 min. An aliquot was taken from the top layer after separation in a centrifuge and filtered through a 0.2 µm PVDF filter.

An HPLC coupled with a PDA detector (Agilent Technologies HPLC 1200 Series, Santa Clara, CA, USA) was used to determine the polyphenolic composition of methanolic extracts of prepared oils following the method described by Panić et al. [32] with minor modifications. The amount of injected extract was 5 μL. Separation of phenolic compounds was performed on a Phenomenax C18 column (Kinetex 150 mm × 4.6 mm, 2.6 μm, 100 Å, Torrance, CA, USA) at 30 °C. The gradient programs used for the analysis were 0.1% formic acid solutions in water (mobile phase A) and 0.1% formic acid solutions in methanol (mobile phase B). At 0 to 3 min, the gradient was 90% A and 10% B. From 3 to 15 min, the percentages changed from the starting values to 50% A and 50% B, and from 15 to 20 min they changed to 40% A and 60% B. From 20 to 25 min, the gradient changed to 0% A and 100% B for elution, and remained there until 26 min. From 26.1 min, the percentages returned to the initial values, where they remained until 28 min. The flow rate was 0.9 mL/min during the entire analysis. The phenolic compounds were identified by comparing their retention times and their UV spectra with those of gallic acid, 3,4-dihydroxybenzoic acid, tyrosol, 4-hydroxybenzoic acid, secoisolariciresinol diglucoside (SDG), chlorogenic acid, vanillic acid, 2,4-dihydroxybenzoic acid, caffeic acid, syringic acid, vanillin, syringaldehyde, *p*-coumaric acid, ferulic acid, sinapic acid, *o*-coumaric acid, oleuropein, and *trans*-cinnamic acid. Phenolic compounds were quantified using calibration curves prepared from the corresponding standard solutions. Unidentified compounds, as well as content of rosmarinic acid, were quantified by using a gallic acid calibration curve.

### 2.6. Determination of Antioxidant Activity in Oil Samples with the 2,2′-Diphenyl-1-picrylhydrazyl (DPPH) Method

Radical scavenging activity was determined by the method described by Kalantzakis et al. [33], and was defined as the ability of oil to quench the stable 2,2-diphenyl-1-picrylhydrazyl radical (DPPH). Reduction of the amount of free DPPH radical was determined spectrophotometrically by measuring the color change of the reaction solution at 515 nm. Briefly, 10% (*w*/*v*) oil solution in ethyl acetate (1 mL) was added to freshly prepared 0.125 mM DPPH solution (4 mL). The reaction mixture was shaken, and the absorbance was measured at the very beginning of the reaction and after 30 min. The reaction was carried out at the room temperature in the dark. The absorbance was measured against the blank solution (without DPPH radical). The DPPH concentration was calculated from calibration curve, and was used for calculating radical-scavenging activity following Equation (1):

(1) $$\%\;\mathrm{DPPH}\;\mathrm{reduction}=100\times (1-\frac{{\left[\mathrm{DPPH}\right]}_{0}}{{\left[\mathrm{DPPH}\right]}_{30}})$$

where [DPPH]_0_ and [DPPH]_30_ are concentrations of DPPH in the control sample (*t* = 0 min) and in the test mixture (*t* = 30 min of reaction).

### 2.7. Microbiological Analysis of Oil Samples

All microbiological testing was performed according to the prescribed standard ISO methods.

Plate Count (PCA) agar, Sabouraud agar, Violet Red Bile Glucose (VRBG) agar, Half Fraser broth, Fraser broth, Listeria selective agar (Oxford agar), Agar Listeria according to Ottaviani and Agosti (ALOA) agar, and Casein Soy Bean Digest Agar (CASO) agar from Merck (Darmstadt, Germany) were used as culture media and broths for isolation and determination of microorganisms.

The isolation and identification of *Listeria monocytogenes* was performed according to ISO 11290-1 [34]. The samples (25 mL) were transferred to 225 mL of sterile Half Fraser broth, and mixed well to obtain 10-fold dilution, then 0.1 mL of Half Fraser broth cultured at 30 °C for 24 ± 3 h was aseptically inoculated into 10 mL of Fraser broth. The culture was incubated at 37 °C for 48 ± 3 h. A loopful cultured Fraser broth was streaked onto Oxford and ALOA agar and incubated at 37 °C for 24 ± 3 h.

In addition, the determination of the viable total number of microorganisms by inoculating the sample on a culture media incubated at 30 °C was performed in accordance with the ISO 4833 method [35]. Determination of total number of isolated microorganisms is expressed as CFU/mL.

Further, the procedure for enumeration of viable osmophilic yeasts and xerophilic molds in oil samples was performed using solid medium incubated at 25 °C according to ISO 21527-2 [36] with minor modifications. Sabouraud agar was used instead of DG18 agar, and inoculated plates were incubated at 25 ± 1 °C for 5 days.

According to the ISO 21528-2, detection and enumeration of viable bacteria from the *Enterobacteriaceae* family was performed by inoculating the sample on selective Violet Red Bile Glucose agar (VRBG agar) [37]. After 24 ± 2 h of incubation at 37 ± 1 °C, typical grown colonies of *Enterobacteriaceae* that were red or pink in color were counted. In plates with enterobacterial growth, 5 typical colonies were isolated on CASO agar (37 °C/24 ± 2 h), and biochemical confirmation was made. Biochemical confirmation was performed using oxidase assay (Merck, Germany) and API 20 E (BioMérieux, France), which are commercially available, and the procedure was done according to the manufacturers’ instructions.

### 2.8. Sensory Analysis of Oil Samples

For sensory evaluation of the prepared samples, training of the examiners was first performed. A group of 14 examiners passed several tests designed by Meilgaard et al. [38] on model solutions and on freshly produced flaxseed oil. The training process was customized only to bitterness, which was necessary for this part of the research. The selection of panel group members included the following tests: method of investigative sensitivity of taste [39], triangle test [40], ranking [41], and sequential analysis [42]. Eight candidates were selected, who approached the evaluation of coded flaxseed oil samples with and without additions, immediately after sample preparation, and after 2, 4, and 6 months of storage at room temperature. Oil samples (15 mL) were served to the panelists at room temperature, and water and unsalted bread were given to rinse their mouths between samples. Sensory analyses were performed on the principle of sequencing test—“Ranking” according to ISO 8587 [41], and the panelists had to rank the oil samples according to the intensity of bitterness. The oil with the strongest bitterness was associated with number 1, and the least bitter oil with number 6. Scores given by all of the analysts were summed for each oil.

### 2.9. Statistical Analysis

The data obtained in this study are presented as arithmetic means (SV) ± standard deviation (SD) of the mean. Using the Kruskal–Wallis test, all data were analyzed using the statistical program STATISTICA 13.1 at a level of significance *p* < 0.05 (StatSoft, Tulsa, OK, USA).

## 3. Results and Discussion

Cold-pressed flaxseed oil is well known for its health benefits, especially thanks to its high content of essential omega-3 α linolenic acid (ALA), which unfortunately also makes it susceptible to oxidation [2,43]. For this reason, the aim of this paper was to evaluate the influence of different spices and herbs on the oxidative stability of flaxseed oil, as well as the microbial safety, and to test the bitterness intensity of samples by sensory testing with regard to the addition of different spices and herbs.

### 3.1. Oil Quality

During storage of flaxseed oil, various changes are possible that affect its quality, such as hydrolytic degradation and oxidation [3]. Formed degradation products give an unpleasant odor and taste to the oil, while some of them, such as peroxides, are harmful to health. The content of free fatty acids (FFA) and peroxide value (PV) were determined from the basic quality indicators.

#### 3.1.1. Content of Free Fatty Acids (FFAs)

**Figure 1:**
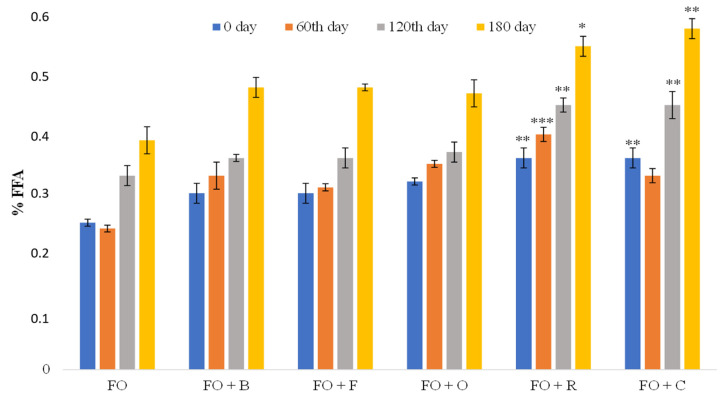
Content of free fatty acids (FFAs) in oil samples before and during the 180th days of storage. Data are presented as the mean ± SD. *Significantly different from the flaxseed oil (* *p* < 0.05; ** *p* < 0.01; *** *p* < 0.001). Abbreviations: FO—flaxseed oil; FO + B—flaxseed oil infused with basil; FO + F—flaxseed oil infused with fennel; FO + O—flaxseed oil infused with oregano; FO + R—flaxseed oil infused with rosemary; FO + C—flaxseed oil infused with chili.

FFA content represents the free fatty acid content expressed as % of oleic acid, which is dominant in most vegetable oils. The content of free fatty acids is an indicator of hydrolytic changes in oil. Hydrolytic changes are associated with the hydrolysis of the glyceride moiety and result in an increase in total fatty acids, and a change in taste is caused by certain free fatty acids [44]. The occurrence of hydrolytic degradation in the oil itself is minimized if the oil production process is done well. Hydrolytic deterioration occurs most often in the seed itself, therefore the content of free fatty acids can be considered as an indicator of seed health [5]. Analyses for FFAs were performed at baseline, and after 2 months, 4 months, and again after 6 months, and the results were compared with the prescribed values of the national regulations on edible oils and fats in the category of cold-pressed oils (because quality parameters are not defined for infused oils), and with the Codex Alimentarius Commission standard for cold-pressed oils (Figure 1).

From the values at the beginning, as well as during the storage, it can be seen that the content of free fatty acids was higher in infused oils compared to flaxseed oil, especially in flaxseed oil with rosemary and chili. Compared to the flaxseed oil, flaxseed oil with rosemary had a statistically significantly higher value of free fatty acids at baseline (0.36 ± 0.017 vs. 0.25 ± 0.006), and after 60 days (0.40 ± 0.012 vs. 0.24 ± 0.006), 120 days (0.45 ± 0.012 vs. 0.33 ± 0.017), and 180 days (0.55 ± 0.017 vs. 39 ± 0.023) of storage. Similar statistically significant higher values were obtained for flaxseed oil with chili: 0.36%, 0.33%, 0.45%, and 0.58%, respectively (Figure 1). Other herbs in flaxseed oil during storage showed a certain increase in these values compared to flaxseed oil, but without statistical significance (*p* > 0.05). According to our results, the higher values of FFAs in infused oils compared to flaxseed oil can be attributed to hydrolytic microbiological changes, since spices and herbs can introduce potential biological hazards, as well as a certain proportion of water in the leaves, which lead to increased enzymatic activity, thus inducing lipolytic reactions in flaxseed oil. In addition, herbs and spices, which are rich in phenolic constituents, can act as pro-oxidants, whose pro-oxidative activity is related to their metal-chelating ability, reducing properties, and radical-scavenging, and thus promote hydrolytic processes [45].

However, although there was an increase in the content of free fatty acids in infused oils after the 180th day of storage, not a single infused oil had a content greater than 2% of FFAs (Figure 1), which was in line with the national regulations on edible oils and fats [46], or 4 mg KOH/g oil according to the Codex Alimentarius Commission standard for cold-pressed oils [47].

The obtained results were similar to those of Almeida et al. [48] and Škevin et al. [28], who demonstrated that FFA values increased during prolonged oil storage. FFA values in these papers also were higher for olive oil with the addition of rosemary compared to pure olive oil, which is consistent with the results obtained in this paper.

#### 3.1.2. Changes in Peroxide Value (PV)

**Figure 2:**
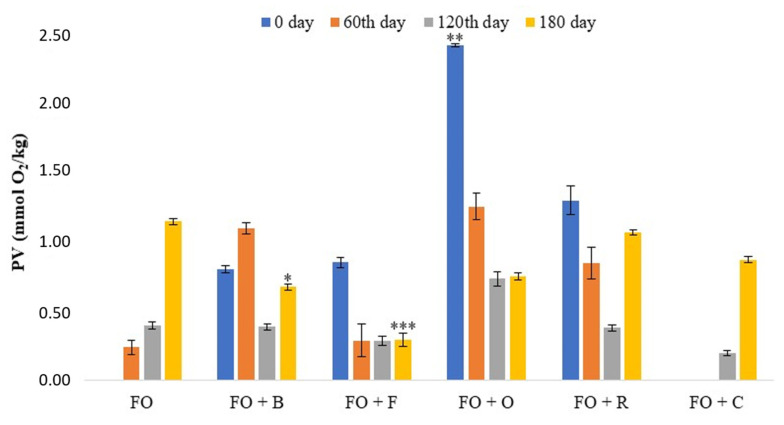
Peroxide value (PV) of oil samples before and during the 180th days of storage. Data are presented as the mean ± SD. *Significantly different from the flaxseed oil (* *p* < 0.05; ** *p* < 0.01; *** *p* < 0.001). Abbreviations: FO—flaxseed oil; FO + B—flaxseed oil infused with basil; FO + F—flaxseed oil infused with fennel; FO + O—flaxseed oil infused with oregano; FO + R—flaxseed oil infused with rosemary; FO + C—flaxseed oil infused with chili.

Apart from hydrolytic deterioration characteristic for seeds, oxidation deterioration is most commonly found in oil as a result of the oil’s exposure to air and other pro-oxidants such as temperature, light, ionizing radiation, and heavy metals, especially copper and iron. Oxidative degradation of fatty acids results in formation of volatile byproducts: aldehydes, methyl ketones, alcohols, acids, ether, esters, and hydrocarbons, which give an unpleasant odor of rancidity to the oil, and have a significant effect on the nutritional value of the oil [4,49,50]. Determination of the degree of fat oxidation by peroxide value provides an assessment of the quality of the fat and, consequently, the ability of its application and storage, since it serves to test the primary products of fat oxidation. Figure 2 shows the results obtained from the peroxide value of flaxseed oil samples and flaxseed oil samples with spices and herbs before and during the storage.

From the obtained data, it can be seen that the primary oxidation products of flaxseed oil were measured barely after 60 days of storage (0.25 mmol O_2_/kg); i.e., the peroxide value increased with the storage. The peroxide value of flaxseed oil increased with the oil storage due to formation of advanced oxidation products, and after 180 days it was 1.6 mmol O_2_/kg (Figure 2).

On the other hand, the results showed that the peroxide value was increased in all oil samples with the addition of the herbs, while in flaxseed oil with chili, which is a spice, it was not increased. The peroxide values of infused oils at the baseline were 0.82, 0.87, 2.46, and 1.32 mmol O_2_/kg for FO + B, FO + F, FO + O, and FO + R, respectively, where a statistically significant higher value was found for FO + O. Peroxides, which are initially present in the oil, can be produced by enzymatic oxidation. By comparing the initial peroxide values, it can be seen that the PV values decreased after 60 days for all herbs except basil, the value of which began to decrease only after four months of storage (PV decreased by 41.46%). Peroxide values were lowest during the fourth month of the storage, although they were almost the same after 180 days of storage for flaxseed oil with oregano and fennel, which had a statistically significant lower value than flaxseed oil on the 180th day of storage. Flaxseed oil with fennel had the lowest values during the entire storage (0.87, 0.30, 0.29, and 0.30 mmol O_2_/kg), followed by flaxseed oil with basil (which also had a statistically lower value on the 180th day of storage), oregano, and rosemary. Further, PV of flaxseed oil with chili grew similarly to flaxseed oil, but much slower; the primary products were measured barely after 120 days of storage, and amounted to 0.89 mmol O_2_/kg. A possible explanation for this is precisely the difference in chemical composition between herbs and this spice, which is especially rich in essential oils.

As expected, during the storage, the peroxide values were lower in the infused oils as compared to the flaxseed oil (Figure 2), which was in line with the literature reported by Gambacorta et al. [51]. The reason for the increased PV in infused oils at the beginning of storage may be due to the higher content of primary oxidizing compounds resulting from the prolonged maceration time. However, the addition of spices and herbs also contributed to the enrichment of flaxseed oil with antioxidant compounds that restricted the formation of hydroperoxide and thus slowed oxidation during storage, and our results were consistent with those obtained in [28] and [52]. According to numerous papers, the addition of spices and herbs to oils resulted in stabilization because the compounds that turned into oils were mainly of phenolic character and possessed antioxidant properties [34,53,54,55,56,57,58].

As can be seen, infused oils exhibited better stability as compared to flaxseed oil, and showed the radical-scavenging efficiency of herbs and spices. PV in all infused flaxseed oil samples ranged between 0 and 2.45 mmol O_2_/kg, indicating a very low degree of oil oxidation. The obtained values were significantly lower than 7 mmol O_2_/kg; i.e., 14 meq O_2_/kg oil, the limit values prescribed by the national regulations on edible oils and fats [46] and the Codex Alimentarius Commission standard for cold-pressed oils [47]. Depending on the obtained results, we could conclude that the natural antioxidants and their synergistic/antagonistic action were suitable in their function for increasing oxidative stability; i.e., the shelf-life of flaxseed oil.

### 3.2. Content and Composition of Polyphenols and DPPH Radical-Scavenging Activity of Oil Samples

Phenolic compounds of spices and herbs offer better antioxidant stability, and contribute to the nutritional value, sensory properties, and prolongation of the oil’s shelf life. They play an important role in human health due to their anti-inflammatory, antiallergic, antimicrobial, antitumor, and antiviral activities [24,59,60]. The content and composition of phenolic compounds in the flaxseed oil samples infused with spices and herbs are shown in Table 1 at the beginning of storage, and after 120 and 180 days of storage.

**Figure 3:**
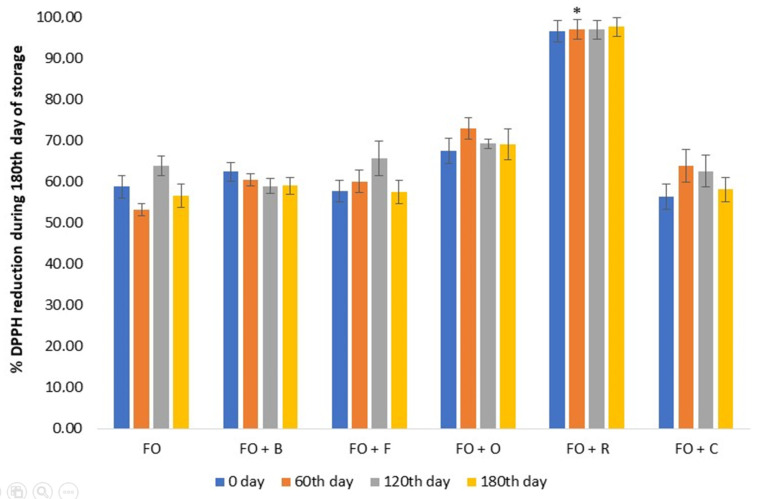
Radical-scavenging activity of oil samples before and during storage. Data are presented as the mean ± SD. *Significantly different from the flaxseed oil (* *p* < 0.05). Abbreviations: FO—flaxseed oil; FO + B—flaxseed oil infused with basil; FO + F—flaxseed oil infused with fennel; FO + O—flaxseed oil infused with oregano; FO + R—flaxseed oil infused with rosemary; FO + C—flaxseed oil infused with chili.

It was evident that phenols were present in small amounts in the tested oil samples, and a larger content was present in the oil samples infused with herbs, especially in flaxseed oil with the addition of rosemary, basil, and oregano. At the beginning of the preparation of infused oils, the phenol content was 5.44 mg/kg for FO, 9.17 mg/kg for FO + F, and 6.81 mg/kg for FO + C, while the statistically significantly higher content was in oils with the addition of rosemary (22.92 mg/kg), basil (22.88 mg/kg), and oregano (19.68 mg/kg). The most abundant phenolic compound was rosmarinic acid in flaxseed oil samples with basil (6.89 ± 1.99 mg/kg), rosemary (5.57 ± 0.02 mg/kg), and oregano (6.8 ± 1.05 mg/kg), followed by SDG, vanillin, vanillinic acid, and *trans*-cinnamic acid. Flaxseed oil results showed that only unidentified compounds were present. Although mostly unidentified compounds were found (Table 1), we also found that there was a positive correlation with the results obtained between antioxidant activity and phenolic compounds (Figure 3, Table 1) such as vanillin, SDG, vanillic acid, rosmarinic acid, *trans*-cinnamic acids, and total phenols. After 180 days of storage, the phenol content decreased by 49.87% (FO + B), 23.12% (FO + F), 48.42% (FO + O), 67.45% (FO + R), and 7.64% (FO + C) compared to the beginning, because phenol degradation occurred due to oxidation reactions (Figure 2). These results coincided with the available literature data, which indicate that phenol degradation occurred during storage at room temperature, and that the degree of degradation depended on the concentration, chemical characterization of individual phenolic components, storage conditions, higher enzyme activity, and presence of reactive oxygen species (ROS), particularly hydroxyl radical [61,62].

To assess the antioxidant activity respective to resistance to oxidative changes of infused oil, we used the most common method—the DPPH method. When DPPH reacts with the natural antioxidants present in flaxseed oil and added herbs and spices, which can donate hydrogen, it is reduced. The antioxidant activity results of the oils are shown in Figure 3.

The DPPH radical-scavenging activity of the studied oils during the entire storage ranged from 56.40% to 97.66%. The antioxidant activity of flaxseed oil with rosemary was highest at the beginning and at the end of the storage compared to the other flaxseed infused oils (97.66%, Figure 3). Flaxseed oil with oregano also had a higher antioxidant value compared to the other oils (69.12%). Pure flaxseed oil had the lowest antioxidant activity (56.70%), while flaxseed oil with fennel had a slightly lower value (57.49%) than the other infused oils.

An increase in antioxidant values in all infused oils occurred during the second and fourth months of storage due to extraction of phenolic compounds from the remaining sludge of spices and herbs in flaxseed oil, as well as the oil itself, but at the end of storage there was a slight decrease in these values (Figure 3). Antioxidant values for flaxseed oil infused with rosemary had a slight increase over the initial value (96.65% vs. 97.66%). Analysis of the results showed that flaxseed oil with rosemary had a significant effect on the antioxidant activity at the 60th day of storage in comparison to flaxseed oil (*p* < 0.05), which resulted from high content or synergistic activity of antioxidant compounds in this oil, while other herbs and spices had no significant effect on the antioxidant activity of pure flaxseed oil (*p* > 0.05). In addition, the results in Table 1 show that flaxseed oil with rosemary contained considerable amounts of phenols (22.92 mg/kg). Flaxseed oil with rosemary had the highest antioxidant activity compared to other oils, because carnosic acid and carnosol are responsible for 90% of its antioxidant properties, and inhibit lipid peroxidation and scavenge free radicals (good scavengers of peroxyl radicals). Its antioxidant properties are also contributed by the isoprenoid quinones, which act as chelators of free radicals and as chain terminators of free radicals [14,63].

Comparing our results using a modified antioxidant activity test method with those of Siger et al. [64], significantly higher values of antioxidant activity were obtained for pure flaxseed oil: 53–64% DPPH vs. 19.30 ± 2.10% DPPH, because the choice of solvent can greatly affect the results of the analysis. Schwarz et al. [65] stated a “polar paradox”, according to which lipophilic antioxidants exhibit better activity in a polar solvent, whereas polar antioxidant activity is more pronounced in a lipophilic medium.

### 3.3. Microbial Safety of the Oil Samples

Despite all mentioned beneficial effects, spices and herbs can lead to human health hazards due to present microorganisms. Spices and herbs, plant products, cereals, and oilseeds can be contaminated with pathogenic bacteria, and microbiological testing was carried out to ensure microbial food safety of the product. In accordance with the Guideline on microbiological food criteria of the Ministry of Agriculture of the Republic of Croatia [66], for edible vegetable and animal fats and oils, a microbiological examination was carried out for: aerobic mesophilic bacteria, bacteria from the *Enterobacteriaceae* family, *Listeria monocytogenes,* and yeasts and molds. The results of microbiological examination are shown in Table 2.

According to the Guideline [66], up to 10 CFU/mL is allowed for edible vegetable and animal fats and oils (m = 10 CFU/mL) for aerobic mesophilic bacteria, bacteria from the *Enterobacteriaceae* family, and yeasts and molds, while *Listeria monocytogenes* should not be isolated in edible oil samples. The absence of *Listeria monocytogenes* is also a food safety criteria prescribed by the Commission Regulation on microbiological criteria for foodstuffs [67]. From the results obtained at the beginning of the storage, we can see that all samples except flaxseed oil with basil corresponded with the legislation requirements [66], and were in the upper limit of tolerance due to the presence of microorganisms (up to 10 CFU/mL); while flaxseed oil with basil, due to the increased amount of the mold colony (56 CFU/mL), was unsatisfactory and was not fit for human consumption.

The obtained results (Table 2) showed that all the samples of the infused oils during storage were in compliance with the Guideline on microbiological food criteria [66] except flaxseed oil with basil. During storage, this oil sample still remained unfit for human consumption, not only because of the increased number of mold colonies as a start, but also because of the increased number of aerobic mesophilic bacteria colonies (80 CFU/mL on the 60th and 120th days of storage), although this number decreased slightly after six months (30 CFU/mL). Herbs possess a very wide spectrum of activity against microorganisms, and their antimicrobial activities vary according to the types of herbs (origin and bioactive compounds). In fact, herb constituents may affect several targets of microorganisms: cell membrane, adhesins, genetic material, and enzymes, and are able to disintegrate the outer membrane, releasing LPS and increasing the permeability of the cytoplasmic membrane. In this way, herbs and spices may impact the biofilm production and motility of bacteria [12,20]. In addition, some molds can be dangerous and produce toxins such as aflatoxins and ochratoxins, which are carcinogens, teratogens, and mutagens. Ochratoxins are produced by various species of *Aspergillus* and *Penicillium* molds and are the most toxic, while *Aspergillus flavus*, *A. parasiticus*, and *A. nomius* produce aflatoxin [68,69]. Molds produce the enzyme lipase and spores that help them to survive the anaerobic nature of the oil. In addition to our results for flaxseed oil with rosemary, the presence of microorganisms in this oil was not found, although a small number of mold colonies were found after the fourth (1 CFU/mL) and sixth months (3 CFU/mL) of storage. The possible reason may be that the spores from the air contaminated the sample during analysis. The obtained results for this oil were expected, since rosemary has higher content of phenolic compounds and higher antimicrobial and antioxidant activities due to numerous antioxidants, in particular carnosolic acid (97.66% DPPH, Figure 3) [14,24,63]. Overall, the antimicrobial action of herbs and spices was not the result of the action of a single compound, but of the synergistic effect of multiple components. Plants with many different mechanisms can prevent the attack of microbes on their tissues, especially with antimicrobial polyphenolic polymer complex (lignin). Lignin is specifically resistant to microbial degradation, as was evident from the results of flaxseed oil with rosemary, and to a considerable measure for other oils. Flaxseed oil samples with fennel and chili showed an increase in the number of microorganisms (CFU/mL) during the fourth (fennel: 10 for aerobic mesophilic bacteria and 7 for molds; chili: 10 for aerobic mesophilic bacteria) and sixth (fennel: 4 for aerobic mesophilic bacteria and 6 for molds; chili: 10 for aerobic mesophilic bacteria and 5 for molds) months of storage, which can be correlated with the results of the peroxide number (Figure 2), as its increase during the indicated storage months was observed. Since the peroxide value indicates the degree of oxidation of the oil, and since the oxidation process produces free radicals such as hydroperoxide, in this way, the availability of water molecules in the oils was favorable to the growth of microorganisms. Therefore, another physical factor responsible for triggering the deterioration of oil is the content of available water and pH. Cereals, legumes, nuts, and oilseeds generally dry out after harvest, so low water activity limits the growth of microflora, causing spoilage of xerophytic and xerotolerant microscopic fungi [70]. However, the microflora may vary depending on the type of herbs or spices, the environmental conditions, the season, and the soil in which the spices and herbs grew. The way spices and herbs are stored will often affect the subsequent growth of particular groups of microbes.

The results obtained by microbiological testing of the oil (Table 2) were consistent with Ciafardini et al. [71], who investigated the presence of microorganisms in infused olive oils. This analysis, as well as our data, confirmed the hypothesis that different types of spices and herbs in oil can affect the survival of microorganisms through habitat alteration. Furthermore, although yeasts were present in the literature references in some samples, the presence of yeasts was not found in the oil samples of this study.

In addition, the findings were consistent with the results of other authors, who noted that spices and herbs could be contaminated due to the conditions in which they are grown and harvested, and that microorganisms were less present in spices and herbs with higher antimicrobial activity [72,73], which was the case of flaxseed oil with rosemary. The results of present study indicated that flaxseed oil with oregano had the maximum allowed number of colonies for aerobic mesophilic bacteria (10 CFU/mL), as well as bacteria from the *Enterobacteriaceae* family (10 CFU/mL), while the number of mold colonies (5 CFU/mL on the 60th, and 4 CFU/mL on the 120th and 180th days of storage) was slightly lower. It was also evident from the results that there was no reduction in the number of microorganisms during storage, from which we can conclude that the oregano was already contaminated with an increased number of microorganisms before its addition to flaxseed oil, despite the results in Figure 3 (69.12% DPPH) indicating an increased antioxidant activity of oregano. These results correspond in part to the literature, since there are no data on the number of colonies of bacteria from the *Enterobacteriaceae* family but on coliform bacteria [74], but the presence of molds and aerobic mesophilic bacteria was in accordance with literature. Microbiological testing of bacteria from the *Enterobacteriaceae* family is included in the Guideline on microbiological food criteria [66] as an indicator of hygiene. Their presence and increased numbers can indicate unhygienic conditions during preparation, processing, packing, and market display of oils.

In comparation to Ciafardini et al. [71], our results for flaxseed oil with chili showed the presence of aerobic mesophilic bacteria and molds during storage, but with a lower extent of contamination (10 CFU/mL for aerobic mesophilic bacteria on the 120th and 180th days of storage, Table 2). Likewise, the initial number of bacteria from the *Enterobacteriaceae* family was inhibited during storage, which was due to the antioxidants present, as shown by the results in Figure 3. The increase in the number of the microorganisms was observed at the fourth and sixth months of storage (Table 2), which could also be related to the results of the peroxide value (0.88 mmol O_2_/kg on the 180th day of storage, Figure 2) due to the availability of available water. The results of the analyses in the literature on commercial edible oils and infused oils, as well as our results, showed that certain spices or herbs and their concentration in oil strongly affected the survival of microorganisms, regardless of the type and amount of initial contamination [74,75].

Aware of the biological hazards that certain spices and herbs can bring to oils, new methods of inactivating microorganisms need to be developed in order to ensure microbial food safety, including those that allow the positive properties of spices and herbs to be preserved, such as phenolic compounds that contribute to antioxidant stability. Until now, γ irradiation was applied to samples that had an increased number of microorganisms, so there is a need to develop new methods or improve existing ones that aim to preserve nutritional and sensory values.

### 3.4. Sensory Properties of Oil Samples

Sensory analysis of flaxseed infused oil was done to assess whether the added spices and herbs would further increase the bitterness of flaxseed oil, as its bitterness increases during storage of cold-pressed flaxseed oil due to cyclolinopeptide E biosynthesis [76] For this reason, bitterness was chosen to monitor the sensory characteristics of the oils during storage. The only oil that had a fully developed sensory analysis for now is virgin olive oil. For other oils, no sensory analysis has been developed to the same extent as for virgin olive oil, although sensory indicators are a very important part of oil quality. Based on the above, a panel was selected to include people who were trained sensory analysts of virgin olive oil (selected and trained according to the methods of the International Olive Council, IOC). In the selected panel of people, training was performed according to the already-described tests. In this study, a sensory analysis of bitterness intensity was performed, in which the oil with the highest bitterness intensity received a note of 1, while the oil with the lowest bitterness intensity received a note of 6. Since flaxseed oil with basil is not fit for human consumption according to microbiological criteria, it was excluded from sensory testing. The results of sensory testing of flaxseed oil infused with spices and herbs during storage are shown in Table 3.

The panelists evaluated flaxseed oil samples with the addition of 5% spices and herbs in the form of macerate, and the results represent the sum of the panelists’ ratings. According to the results of sensory testing, flaxseed oil (control sample) received the highest score due to the lower bitterness intensity perceived by the panelists after oil preparation (Table 3). However, the results also show that during storage of cold-pressed flaxseed oil at room temperature, its bitterness increases. As mentioned earlier, the component responsible for this phenomenon is cyclolinopeptide E [76], and thus its bitterness among the tested oils was higher.

During the second, fourth, and six months of storage, chili had the lowest bitterness intensity. This spice at that period received the highest score because the bitterness was not of the same intensity as at the beginning of the preparation. This result also can be related to the results of the phenol content, which showed that the phenol content in flaxseed oil with chili was much lower (6.07 mg/kg vs. 19.36 mg/kg in flaxseed oil with basil during 4 months of storage) compared to other infused flaxseed oils whose phenols can also contribute to the bitterness of the oil.

On the other hand, panelists rated rosemary with the lowest scores during 180 days of storage due to its high concentration of phenolic compounds and strong intensity of bitterness (Table 3). Flaxseed oil with fennel and oregano had similar results regarding bitterness intensity. Flaxseed oil with fennel was rated at the beginning of the preparation, as well as flaxseed oil with rosemary. Intense flavor and aroma respective to bitterness of flaxseed oil with oregano, which also had a high phenol content as rosemary, made it more bitter than others (Table 3). Antoun and Tsimidou [77] found the same results if we compare our results of oil with the addition of oregano and rosemary. In their work, olive oil with the addition with oregano was more acceptable to panelists than the addition of rosemary in the same concentration. According to the obtained results, we expect that certain infused oils would be less accepted among consumers precisely because of the strong aroma intensity of the added herbs and spices, and that flaxseed oil with chili might be well accepted by consumers. The reason for this could be obtained higher scores, lower phenol content, and knowledge of this spice, its taste, and its use in the preparation of meals. Chili is among the oldest cultivated plants in the world and is used as a spice to flavor dishes worldwide It is appreciated for its sensory attributes of aroma, color, and pungency. In addition, it has ethnomedicinal prestige and is used to treat a variety of human illnesses. Rosemary is also often used in Mediterranean cuisine, but may be more acceptable to consumers if it was added to the oil at a lower concentration.

Furthermore, it should also be noted that the results of the sensory testing might have been different if flaxseed oil with basil had been included in the testing, but it was excluded due to the increased number of microorganisms. The obtained results of the sensory analysis of the infused oils were significant, because the intensity of bitterness can affect consumers’ acceptance of the product.

## 4. Conclusions

The addition of spices and herbs in flaxseed oil affected the hydrolytic changes in the oil, but were far less than 2% of FFAs, which is in line with the regulations on edible oils and fats, and improved its storage stability in terms of its highest antioxidant potential, higher content of total phenols, as well as decreased peroxide value. On the other hand, the sensory analysis showed that some spices and herbs had a strong bitterness intensity at a concentration of 5%, but flaxseed oil with chili had the lowest bitterness intensity. Results of this study have important food-processing applications and could definitely create an impact in the market, as there was no adverse health effect on the usage of natural antioxidants. Furthermore, microbiological examinations have shown that flaxseed oil samples with spices and herbs and a pure flaxseed oil correspond the microbiological criteria of the Guideline on microbiological food criteria, except for flaxseed oil with basil. These results indicated a necessity to develop new methods, in addition to existing ones, that would inactivate the microorganisms in heterogeneous samples such as spices and herbs, as well as in oils, and that should also preserve the nutritional values and beneficial properties of the product.

## Figures and Tables

**Table 1:** Phenolic compounds content in oil samples.

Polyphenol (mg/kg)	FO			FO + B			FO + F			FO + O			FO + R			FO + C		
	0 Day	120th Day	180th Day	0 Day	120th Day	180th Day	0 Day	120th Day	180th Day	0 Day	120th Day	180th Day	0 Day	120th Day	180th Day	0 Day	120th Day	180th Day
Vanillic acid	nd	nd	nd	nd	nd	nd	nd	nd	nd	nd	nd	nd	3.47 ± 0.04	nd	nd	nd	nd	nd
Vanillin	nd	nd	nd	5.54 ± 0.01	nd	nd	nd	nd	nd	nd	nd	nd	nd	nd	nd	nd	nd	nd
Rosmarinic acid	nd	nd	nd	6.89 ± 1.99	nd	nd	nd	nd	nd	6.8 ± 1.05	5.87 ± 0.03	nd	5.57 ± 0.02	5.86 ± 0.03	nd	nd	nd	nd
SDG	nd	nd	nd	nd	nd	nd	nd	nd	nd	nd	nd	nd	5. 55 ± 0.05	nd	nd	nd	nd	nd
*trans*-cinnamic acid	nd	nd	nd	2.89 ± 0.13	nd	nd	nd	nd	nd	nd	nd	nd	nd	nd	nd	nd	nd	nd
un.c.	5.4 ± 0.31	5.62 ± 0.31	10.58 ± 4.9	7.56 ± 3.86	7.54 ± 4.63	11.4 ± 9.26	9.17 ± 5.10	8.74 ± 6.19	7.05 ± 1.54	12.88 ± 13.3	13.49 ± 15.7	9.53 ± 4.77	8.33 ± 2.92	8.86 ± 4.05	7.46 ± 4.43	6.81 ± 2.05	6.07 ± 1.19	6.29 ± 1.11
Total polyphenols (mg/kg)	5.44	5.62	10.58	22.88 **	7.54	11.47	9.17	8.74 *	7.05 *	19.68 *	19.36 ***	9.53	22.92 ***	14.72 **	7.46	6.81	6.07	6.29 *

Flaxseed oil samples were prepared with the addition of 5% spices and herbs in the form of macerate. Data are presented as the mean ± SD. *Significantly different from the flaxseed oil (* *p* < 0.05; ** *p* < 0.01; *** *p* < 0.001). Abbreviations: FO—flaxseed oil; FO + B—flaxseed oil infused with basil; FO + F—flaxseed oil infused with fennel; FO + O—flaxseed oil infused with oregano; FO + R—flaxseed oil infused with rosemary; FO + C—flaxseed oil infused with chili; nd—nondetectable; un.c.—unidentified compounds.

**Table 2:** Number of microorganism colonies in oil samples before and during storage.

Experimental Oils ^a^	*Enterobacteriacae* (CFU/mL)				Aerobic Mesophilic Bacteria (CFU/mL)				Molds (CFU/mL)				*Listeria monocytogenes* (CFU/mL)			
	0 Day	60th Day	120th Day	180th DAY	0 Day	60th Day	120th Day	180th Day	0 Day	60th Day	120th Day	180th Day	0 Day	60th Day	120th Day	180th Day
FO	1	<1	<1	<1	10	<1	5	<1	3	<1	<1	<1	n.d.	n.d.	n.d.	n.d.
FO + B	9	<1	10	<1	10	80	80	30	56	56	60	16	n.d.	n.d.	n.d.	n.d.
FO + F	<1	<1	<1	2	5	2	10	4	2	<1	7	6	n.d.	n.d.	n.d.	n.d.
FO + O	10	<1	10	10	10	10	10	10	2	5	4	4	n.d.	n.d.	n.d.	n.d.
FO + R	<1	<1	<1	<1	<1	<1	<1	<1	<1	<1	1	3	n.d.	n.d.	n.d.	n.d.
FO + C	10	<1	<1	<1	6	8	10	10	1	3	<1	5	n.d.	n.d.	n.d.	n.d.

^a^ Abbreviations: FO—flaxseed oil; FO + B—flaxseed oil infused with basil; FO + F—flaxseed oil infused with fennel; FO + O—flaxseed oil infused with oregano; FO + R—flaxseed oil infused with rosemary; FO + C—flaxseed oil infused with chili; CFU—colony forming units; n.d.—not detected.

**Table 3:** Ranking test—rank sums of bitterness of oil samples before and during storage.

Experimental Oils ^a^	0 Day (Score)	60th Day (Score)	120th Day (Score)	180th Day (Score)	Total Score
FO	32	28	19	19	98
FO + F	22	27	15	24	88
FO + O	26	19	16	24	85
FO + R	22	24	15	21	82
FO + C	23	33	22	37	115

^a^ The results represent the sum of the ratings of all panelists. Abbreviations: FO—flaxseed oil; FO + F—flaxseed oil infused with fennel; FO + O—flaxseed oil infused with oregano; FO + R—flaxseed oil infused with rosemary; FO + C—flaxseed oil infused with chili.

## Data Availability

The original contributions generated for this study are included in the article; further inquiries can be directed to the corresponding author.
